# Polyvinyl Chloride Modified Carbon Paste Electrodes for Sensitive Determination of Levofloxacin Drug in Serum, Urine, and Pharmaceutical Formulations

**DOI:** 10.3390/s21093150

**Published:** 2021-05-01

**Authors:** Fatehy M. Abdel-Haleem, Sonia Mahmoud, Nour Eldin T. Abdel-Ghani, Rasha Mohamed El Nashar, Mikhael Bechelany, Ahmed Barhoum

**Affiliations:** 1 Chemistry Department, Faculty of Science, Cairo University, Giza 12613, Egypt; fatehy@sci.cu.edu.eg (F.M.A.-H.); sonia_mahmoud93@yahoo.com (S.M.); nour@sci.cu.edu.eg (N.E.T.A.-G.); rasha.elnashar@cu.edu.eg (R.M.E.N.); 2 Institut Européen des Membranes, IEM UMR 5635, Université Montpellier, CNRS, ENSCM, 34090 Montpellier, France; mikhael.bechelany@univ-montp2.fr; 3 NanoStruc Research Group, Chemistry Department, Faculty of Science, Helwan University, Cairo 11795, Egypt; 4 National Centre for Sensor Research, School of Chemical Sciences, Dublin City University, Dublin D 09, Ireland

**Keywords:** potentiometric sensors, plasticized carbon paste electrode, coated carbon paste electrode, levofloxacin, levoxin^®^, spiked sample

## Abstract

Levofloxacin (LF) is a medically important antibiotic drug that is used to treat a variety of bacterial infections. In this study, three highly sensitive and selective carbon paste electrodes (CPEs) were fabricated for potentiometric determination of the LF drug: (i) CPEs filled with carbon paste (referred to as CPE); (ii) CPE coated (drop-casted) with ion-selective PVC membrane (referred to as C-CPE); (iii) CPE filled with carbon paste modified with a plasticizer (PVC/cyclohexanone) (referenced as P-CPE). The CPE was formulated from graphite (Gr, 44.0%) and reduced graphene oxide (rGO, 3.0%) as the carbon source, tricresyl phosphate (TCP, 47.0%) as the plasticizer; sodium tetrakis[3,5-bis(trifluoromethyl)phenyl] borate (St-TFPMB, 1.0%) as the ion exchanger; and levofloxacinium-tetraphenylborate (LF-TPB, 5.0%) as the lipophilic ion pair. It showed a sub-Nernstian slope of 49.3 mV decade^−1^ within the LF concentration range 1.0 × 10^−2^ M to 1.0 × 10^−5^ M, with a detection limit of 1.0 × 10^−5^ M. The PVC coated electrode (C-CPE) showed improved sensitivity (in terms of slope, equal to 50.2 mV decade^−1^) compared to CPEs. After the incorporation of PVC paste on the modified CPE (P-CPE), the sensitivity increased at 53.5 mV decade^−1^, indicating such improvement. The selectivity coefficient (${\log\;K}_{{\mathrm{LF}}^{2+},{\mathrm{Fe}}^{+3}}^{\mathrm{pot}.}$) against different interfering species (Na^+^, K^+^, NH^4+^, Ca^2+^, Al^3+^, Fe^3+^, Glycine, Glucose, Maltose, Lactose) were significantly improved by one to three orders of magnitudes in the case of C-CPE and P-CPE, compared to CPEs. The modification with the PVC membrane coating significantly improved the response time and solubility of the LF-TPB within the electrode matrix and increased the lifetime. The constructed sensors were successfully applied for LF determination in pharmaceutical preparation (Levoxin^®^ 500 mg), spiked urine, and serum samples with high accuracy and precision.

## 1. Introduction

PVC membrane electrodes (PVCEs) are one of the subdivisions of potentiometric sensors. They suffer the restriction of using an inner filling solution that causes Donnan failure, long response time, short term stability, solubility restriction of the ionophores within the PVC membrane matrix, and high detection limits, and low mechanical stability for long-term usage [1,2]. Carbon-paste electrodes (CPEs) are a special type of ion-selective electrode (ISEs) that have attracted great attention due to their chemical inertness, robustness, renewability, stable response, low ohmic resistance, no need for an internal solution, offering an easily renewable surface for electron exchange, and suitability for a variety of sensing and detection applications [2,3]. These electrodes are typically made of graphite powder, ionophore for binding the analyte, pasting liquid (mineral oils), and other modifiers that facilitate the mobility and binding of the analytical species in the sample solutions with the electrode active surface, which in turn reduces the response time [4,5]. They are also non-toxic and environmentally friendly electrodes with applicability for voltammetric, amperometric, and potentiometric measurements. The carbon paste of CPEs has been modified by ionic liquid, tetraphenyl borate derivatives and conductive polymers, which is not possible in the case of PVCEs [2,3,6]. These modifications significantly improved the detection limit of the CPEs, intrinsic conductivity, the solvating ability for a wide range of soluble and insoluble ionophores, and linear dynamic range, response time, and other properties [6,7]. Carbonaceous nanomaterials such as graphene (G), graphene oxide (GO), reduced graphene oxide (RGO), multiwalled carbon nanotubes (MWCNTs) were also studied and discussed extensively and comprehensively in the literature [8,9,10]. Their unique properties of high electrical conductivity, high surface-to-volume ratio, and ability to be solvent cast to develop uniform films have shown a great improvement of the sensitivity and detection limit of the CPEs [9,10,11,12].

Levofloxacin (LF) is one of the isomeric racemic mixture forms of ofloxacin [13]. It is used to treat a variety of bacterial infections and belongs to a class of drugs known as quinolone antibiotics. It is a prescription drug that comes as an oral tablet, oral solution, and ophthalmic solution (eye drop). It is commercially available in the form of a tablet, injection, and oral solution. It is also rapidly and essentially absorbed completely after oral administration, with a plasma concentration profile over time that is essentially identical to that obtained from intravenous administration of the same amount over 60 min [14,15,16]. LF exhibits antimicrobial activity of broad-spectrum against Gram-positive and Gram-negative bacteria. It works via inhibition of bacterial topoisomerase IV and DNA gyrase, enzymes required for DNA replication, transcription, repair, and recombination [16]. Thus, LF is commonly used for the treatment of several diseases, especially respiratory, urinary, and skin infections [15]. Side effects of overdose include dizziness, drowsiness, disorientation, slurred speech, nausea, and vomiting, tendon problems, nerve damage, serious mood or behavior changes, or low blood sugar [14,15,16]. In rare cases, LF may cause damage to the aorta, which could lead to dangerous bleeding or death [14,15,16].

To date, numbers of analytical techniques have been reported for LF determination, it included high-performance liquid chromatography (HPLC) [17,18,19,20], capillary electrophoresis (CE) [21,22], UV-vis spectrophotometry [23,24], flow injection analysis (FIA) [25,26], nuclear magnetic resonance (NMR) [27], and enzyme-linked immunosorbent assay (ELISA) [28]. However, most of these methods lack simplicity, applicability for routine analysis, be expensive, require sophisticated instrumentation, multi-step sample preparation, and not appropriate for colored and turbid solutions [29]. Electrochemical determination of LF using potentiometry accounts for these problems. On the other hand, LF has a very important property which is its high pH-dependence, i.e., the presence of the levofloxacin in different forms including zwitterionic (LF^±^), cationic (LF^+^, LF^2+^), and anionic (LF^−^) depending on the medium pH [30,31,32,33], which may affect the potentiometric determination. To date, two potentiometric trials have been reported for LF determination [30,31]. The first reported method depended on the ion-pair formation and exhibited good sensitivity and selectivity, but the existence of LF with different ratios in different forms, as a function of pH change, was not discussed [30]. The second method in [31] discussed this issue, but the lifetime of the sensors was the limiting factor, in addition to the low solubility of the ion pair within different sensors that caused heterogeneity of sensors, and the lower slope.

In this study, new designs of potentiometric CPEs were developed for LF determination with high sensitivity and selectivity. The optimum CPEs were formulated from graphite (Gr), reduced graphene oxide (rGO), tricresyl phosphate (TCP) as a plasticizer, sodium tetrakis[3,5-bis(trifluoromethyl)phenyl] borate (St-TFPMB) as an ion exchanger, and levofloxacinium-tetraphenylborate (LF-TPB) as a lipophilic ion pair. The CPE of the best response characters was modified by the PVC layer coating on the CPE surface (C-CPE), or by incorporating the PVC/cyclohexanone paste as a plasticizer for the carbon paste (P-CPE). The Morf water layer test was used to study the probability of water penetration to the coated electrode surface and relate that to the selectivity. The effect of the pH on the response characteristics of these new designs was studied in detail and related to selectivity, linear dynamic range and detection limit [31]. More interestingly, the constructed sensors were successfully applied for the LF determination in blood serum, urine, and commercial formulations (Levoxin^®^ 500 mg, Cairo, Egypt) with high sensitivity.

## 2. Experimental

### 2.1. Chemicals and Solutions

**Figure 1:**
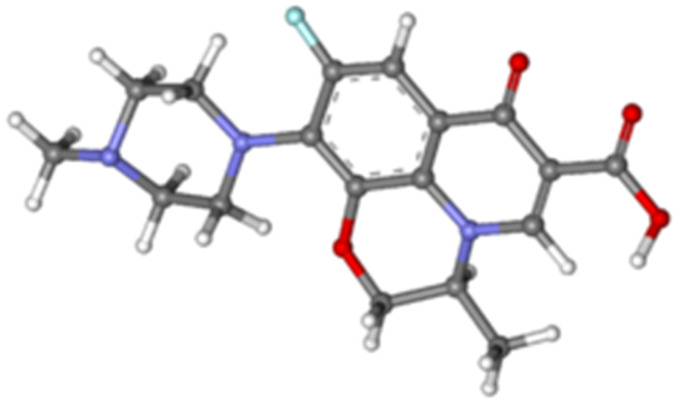
Chemical structure of levofloxacin drug. Nanomenclture: (-) -(S)-9-fluoro-2,3-dihydro-3-methyl-10-(4-methyl-1-piperazinyl)-7-oxo-7H-pyrido[1,2,3-de]-1,4-benzoxazine-6-carboxylic acid hemihydrate. Carbon atoms (gray), hydrogen atoms (silver), oxygen atoms (red), nitrogen atoms (vilot), florine atom (turquoise).

Analytical reagent grade chemicals were used through this work as received with no further purification, and doubly distilled water was used. National Organization for Drug Control and Research (NODCR, Giza, Egypt) provided the authors with Levofloxacin (LF) drug; chemical stureuce of the LF is shown in Figure 1. High molecular weight Polyvinyl chloride (PVC, Mw~43000, Sigma-Aldrich), tetrahydrofuran (THF, 96.5%, Sigma-Aldrich), sodium tetraphenylborate (Na-TPB, 99.9%, Sigma-Aldrich), dioctyl phthalate (DOP, 99.5%, Sigma-Aldrich), graphite (Gr, 99.9%, <45μM, Sigma-Aldrich), tricresyl phosphate (TCP, 98.5%, Sigma-Aldrich) and sodium tetrakis (trifluoromethyl)phenyl borate (St-TFPMB, 99.9%, Sigma-Aldrich), multiwalled carbon nanotubes (MWCNTs, NC7000™, Nanocyl), graphene oxide nanosheets (GO, 15-20 sheets, 4-10% edge-oxidized, Sigma-Aldrich), and β-cyclodextrin (CD, 99.0%, Sigma-Aldrich) were used for electrode preparation. Hydrochloric acid (30%), sodium hydroxide (97%), glucose (99%), maltose (99%), glycine (98) %, lactose (99) %, acetone (95%) acetic acid (99.5%), phosphoric acid (98%), sodium acetate (99%), aluminum sulfate (97%), ferric sulfate (96%), and chloride salts of ammonium (99%), sodium (99%), potassium (99%) and calcium (99%) were obtained from ADWIC (Cairo, Egypt), and were used for preparing standard solutions of the selectivity test. The commercial pharmaceutical preparation Levoxin^®^ (500 mg coated tablet of levofloxacin) was bought from the local market. HCl/KCl buffer of pH 2.2 and Acetate buffer of pH 4.1 were prepared and used for the preparation of (10^−2^ M) LF which was used to prepare the more diluted solutions [34,35]. Reduced graphene oxide (RGO) was prepared by the reduction of GO using NaBH_4_ [36].

### 2.2. Preparation of the LF–TPB Ion-Pair

The ion pair (LF-TPB, levofloxacinium–tetraphenylborate) was prepared by adding 25 mL of 10^−2^ M LF drug to 25 mL of 10^−2^ M Na-TPB drop by drop with constant stirring. The yellow colloidal solution was obtained and then left for a week for coagulation. The solution was then filtered, washed with distilled water, dried at room temperature, and finally ground to a fine powder. The stoichiometry and chemical composition of the obtained precipitated ion-pair (LF-TPB) was identified and ensured by (C, H, N) elemental analysis [31].

### 2.3. Preparation of the PVC Membrane Electrodes

Five PVC membrane electrodes (PVCEs, sensors 1–5) of different component ratios were prepared as reported before [37,38,39]. The PVC membranes were fabricated by dissolving appropriate amounts of PVC powder, lipophilic ion pair (LF-TPB), and plasticizer (CD or St-TFPMB) in 3 mL THF (solvent). The four components were added in terms of weight percentages with a total weight of 0.3 g in a 5-cm diameter Petri dish. A heterogeneous mixture was obtained after the dissolution of all the components that form viscous solution, which was then left for two days for drying in the open air. The membrane was cut into four parts, and each of the four membranes (0.4-mm thickness) was removed carefully from the glass plate and stuck to one end of a “PVC tube” using a PVC/THF slurry and left to dry for 10 min. The electrode was filled then with 10^−3^ M of LF and 10^−2^ M of NaCl as an inner filling reference solution [34,40]. Finally, the same solution was used for soaking for 30 min before use. It is known that the sensitivity, linearity, and selectivity obtained for a given PVCE depends significantly on the membrane composition and nature of the ion pairs and the plasticizer [1]. Thus, the ratio of membrane ingredients, time of contact, the concentration of the equilibrating solution, etc. were optimized after a good deal of experimentation to provide a PVCE, which generates reproducible and reliable stable potential.

### 2.4. Preparation of the Carbon Paste Electrodes

**Figure 2:**
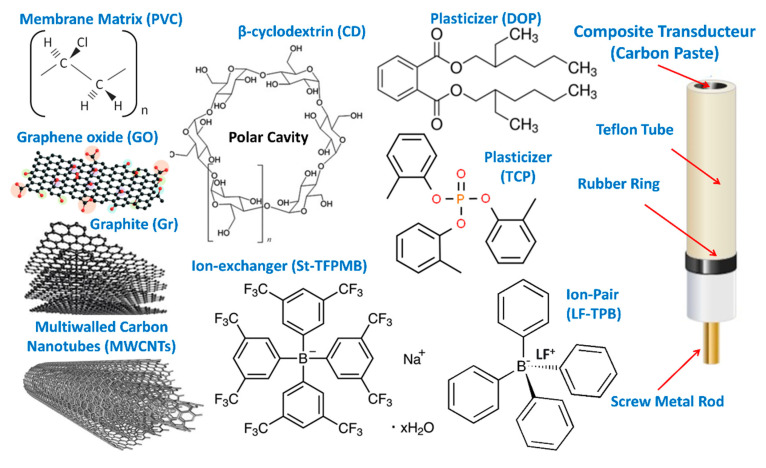
Formulation of the different carbon paste electrodes: carbon paste of CPEs was formulated from different amounts of conductive carbonaceous materials (Gr, GO, rGO, MWCNTs), plasticizers (TCP), ion-exchanger (St-TFPMB), and ion-pair (LF-TPB). The PVC membrane layer of C-CPEs were formulated from (LF-TPB ion-pair, TPB as a plasticizer, TCP or DOP as a plasticizer, and PVC as a polymeric matrix). P-CPEs formulated by mixing carbonaceous materials (Gr), ion-pair (LF-TPB), ion exchanger (St-TFPMB), and plasticizers (TCP, PVC/CD). The electrode compositions (%, *wt*/*wt*) are given in (Table 1).

Six carbon paste electrodes (CPEs, sensors 6–11) were constructed using different amounts of conductive carbonaceous materials (Gr, GO, rGO, MWCNTs), plasticizer (TCP or DOP), St-STFPB ion-exchanger, LF-TPB ion-pair (Figure 2), as previously reported [41,42]. The components of 0.3 g total weight were weighed, mixed for 20 min, and dispersed in the least amount of acetone in an agate mortar, and mixed continually by the pestle to ensure paste homogeneity, Table 1. The carbon paste was packed in a hole (0.35 cm deep, 0.7-cm diameter) at one end of a holder of 12 cm length, which acted as the electrode body. A stainless-steel rod in the center of the holder was included to conduct electricity, and it could be screwed up and down to fill the carbon paste inside the holder and to press the carbon past to obtain a fresh surface of the electrode. A new surface of the electrode can be obtained by turning around the stainless steel rod to compress the paste followed by polishing the surface on a smooth paper; the electrode was soaked in 10^−3^ M LF for 30 min before the first measurement.

### 2.5. Preparation of the PVC Coated Carbon Paste Electrodes

Two PVC membrane-coated carbon paste electrodes (C-CPEs, sensors 12,13) were prepared as follows: 100 µL of a previously prepared PVC membrane cocktail of the composition (5% LF-TPB ion-pair, 1% TPB as a plasticizer, 62.7% DOP as a plasticizer, and 31.3% PVC as a polymeric matrix) was drop-casted onto the surface of an optimized CPE (sensor 11) and was left in the air to dry for 30 min (Figure 2). The C-CPE was finally soaked in a 10^−3^ M LF solution for 30 min.

### 2.6. Preparation of the PVC Plasticized Carbon Paste Electrodes

Four PVC plasticized carbon paste electrodes (P-CPEs, sensors 14–17) were prepared by mixing (Gr, 27.0%), an ion-pair (LF-TPB, 5%), TCP plasticizer (30%), (St-TFPMB, 1.0%) as ion exchanger, (Cyclodextrin (CD), 1.0%) as modifier, and PVC in 9% in cyclohexanone–acetone mixture 1:1 (36%, PVCP) until complete homogeneity (Figure 2). This paste was packed in an insulin syringe that was supplied with copper wire for electrical contact [43]. The P-CPE was finally soaked in a 10^−3^ M LF solution for 30 min.

### 2.7. Electrochemical Measurements

The measurements of both pH and potential (EMF) were recorded using a JANEWAY 3510 pH-meter (Jenway, England). The external reference electrode was a Saturated Calomel Electrode (SCE) (Hanna Instrument, Italy). The potential difference between the two electrodes was measured for 25 mL LF buffered solutions of different concentrations using the following cell assembly at 22 ± 1 °C:

Hg/Hg_2_Cl_2_/KCl (saturated)//sample solution//working electrode

Where one of the PVCEs, CPEs, C-CPEs, or P-CPEs was the working electrode. The calibration graph was obtained using the values of EMF versus the (log (LF)). Scanning electron microscope (ESEM Quanta 450 FEG, Netherlands) with Energy Dispersive X-ray Analyses (EDX) was sued to study the surface morphology of the different sensors.

### 2.8. Morf Test

The water-layer test also called the “Morf Test” was first introduced by Fibbioli et al. to test the potential stability of solid-contact ISEs such as CPEs [44]. This test was performed by recording the potential of the electrode in a solution containing the primary ion (1.0 × 10^−2^ M LF) for one hour, then in a solution containing an interfering ion (1.0 × 10^−2^ M KCl) for one hour, and then changing back to the LF solution again. The Morf test was carried out to test the formation of a thin water layer between the sensing element and the transducer layer which may cause potential drifting [45].

### 2.9. Effect of pH and Selectivity

The change in the potential of a cell comprising the working and reference electrodes was recorded against the pH of the sample solution, which is measured using the glass electrode. The pH was changed by adding very small volumes of 10^−1^ M solutions of HCl or NaOH to 25 mL of 10^−3^ M LF aqueous solution [46]. Additionally, the different response characters as slope, detection limit and linear dynamic range will be tested for the selected sensors at different pH values using the different buffers.

The selectivity coefficients of the different electrodes ($K_{A,B}^{pot}$) were calculated by applying both matched potential method (MPM) and the separate solution method (SSM) [47,48]. In SSM, EMFs of 10^−2^ M of LF and the interfering species *J* in the selected buffer were determined separately as *E*1 and *E*2, respectively. The selectivity coefficient was calculated by Equation (1):

(1) $$K_{LF,J^{z+}}^{pot}=\frac{E2-E1}{S}+\log\;[\mathrm{LF}]-\log\;{\left[J^{Z+}\right]}^{\frac{1}{Z+}}$$

where z+ is the charge of the interfering ion (*J*), and S is the slope of the calibration curve (mV decade^−1^) [47,48].

In MPM, the potential change (Δ*E*) was obtained from adding 10^−2^ M LF solution with a certain amount to LF reference solution (10^−5^ M); addition of 10^−2^ M interfering ion (*J*) to the same LF reference 10^−5^ M LF reference solution was performed to get the same change in potential (). The selectivity coefficient for each interfering ion was calculated by Equation (2):

(2) $$K_{A,B}^{POT}=\frac{a_{1-}a_{2}}{a_{B}}$$

where the activity of LF was increased from *a*_2_ (reference solution) to *a*_1_, *a_B_* is the interferent concentration that caused the same Δ*E* [48].

### 2.10. Practical Evaluation of the Prepared Electrodes

Serum and urine samples were firstly spiked and then 1 mL of the samples (serum/urine) was added a certain amount of pure LF in a 25.0 mL measuring flask for preparation of 10^−3^ and 10^−4^ M solutions [34]. For pharmaceutical samples preparations, three tablets of Levoxin^®^ (500 mg) were ground, weighed, and dissolved in a small volume of the buffer, then the solutions were filtered, then were completed with the same buffers and used for the preparation of (10^−3^ and 10^−4^ M) solutions [34]. These solutions were determined using direct potentiometry using a calibration curve method; recovery was calculated, and statistical analysis was performed to ensure the accuracy and reproducibility of the results.

### 2.11. Statistical Analysis

The standard deviation calculations were based on at least three parallel measurements.

## 3. Results and Discussion

Polyvinyl chloride (PVC) is the world’s third most widely produced plastic synthetic polymer; about 40 million tons are produced each year. It has been also widely investigated as a plastic membrane in the construction of ion-selective electrodes (ISEs). However, its use requires a plasticizer for improving the diffusional mobility of the analysts and electroactive complex [1]. In this study, PVC-modified CPEs have been successfully developed for improved LF detection in serum, urine, and pharmaceutical formulations. To achieve this objective, seventeen different LF-selective electrodes were fashioned for potentiometric determination of the LF drug; PVCEs (sensors 1–5), CPEs (sensors 6–11), C-CPEs (sensors 12,13), and P-CPEs (sensors 14–17). Table 1 lists the percent compositions (*wt*/*wt*%) of the different electrodes (sensors 1–17) and their response properties using acetate buffer at pH 4.1. The best electrode (sensor 16) exhibits a sub-Nernstian slope (53.5 mV Decade^−1^) within the LF concentration range from 1.0 × 10^−2^ M to 1.0 × 10^−4^ M.

### 3.1. Optimization of the PVC Membrane Layer

**Figure 3:**
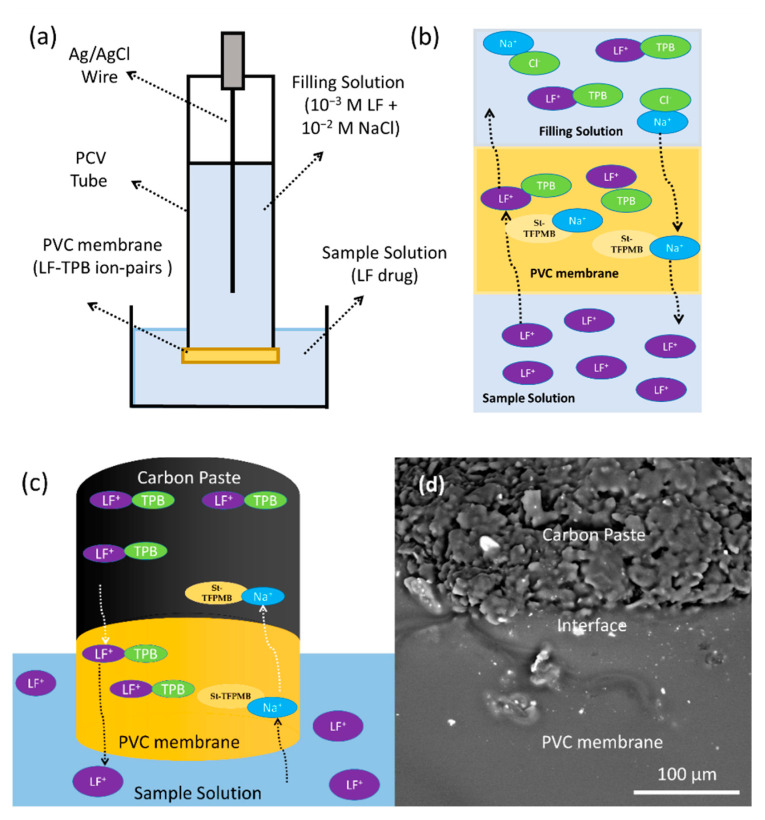
Design of the prepared electrodes and their response characteristics as a potentiometric sensor for LF drug determination at room temperature of 22 ± 2 °C: (**a**) construction of the PVCEs; (**b**) mechanism of ion-pairs exchange between the samples solution, PVC membrane, and filling solution of the PVCEs; (**c**) mechanism of ion-pairs exchange between the samples solution, PVC membrane, and carbon paste of the C-CPEs; (**d**) SEM image of C-CPEs showing the PVC membrane coated on the carbon paste and the interface area between the PVC membrane and the carbon paste.

**Figure 4:**
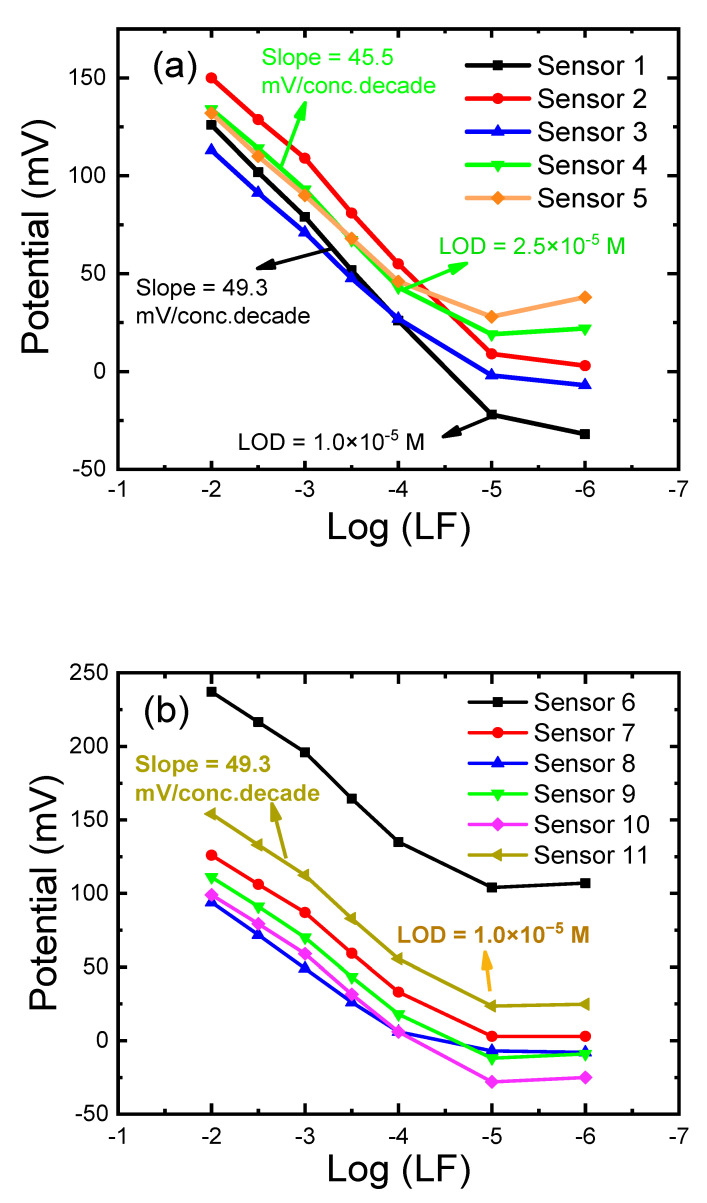
Response characteristics of the prepared electrodes for LF determination: (**a**) calibration curve of PVC membrane electrodes (PVCEs, sensors 1–5) prepared by varying the ratio of PVC powder, ion-pair (LF-TPB), plasticizers (TCP or DOP); ion exchanger (St-TFPMB), modifier (CD), and solvent (THF); (**b**) calibration curve of carbon paste electrodes (CPEs, sensors 6–11) formulated from different carbon sources (Gr, rGO, and MWCNTs), plasticizers (TCP or DOP); ion exchanger (St-TFPMB), and lipophilic ion pair (LF-TPB). The electrode compositions (%, *wt*/*wt*) are given in (Table 1). All measurements were at pH 4.1 using acetate buffer at room temperature of 22 ± 2 °C.

Five PVCEs (sensors 1–5) were prepared and optimized membrane layer responses (linear range of concentration, slope, limit of detection, and response time) by varying the ratio of PVC powder, ion-pair (LF-TPB), and plasticizer (CD or St-TFPMB) to the solvent (THF) (Table 1, Figure 3a). Sensor 1 containing 1.0% St-TFPMB as lipophilic ion-exchanger and 66.0% TCP plasticizer showed a 50 mV decade^−1^ slope, which is higher than sensor 2 prepared with 66.0% DOP as a plasticizer, due to the higher dielectric constant of TCP [1]. However, the solubility of the ion-pair LF-TPB was tested separately in TCP, and it was so limited that it caused the formation of non-homogenous membrane, and so only DOP was tested with the LF-TPB ion-pair in sensors 3–5, Table 1 and Figure 3a. Sensor 3 incorporating only LF-TPB exhibited potentiometric responses of 43.0 mV decade^−1^ in the range of 10^−2^–10^−4^ M, which confirm that all the potentiometric responses in the membranes were only due to the presence of ion-pairs, and the ion-exchange mechanism with the formation of double-layer [49]. The addition of 1.0% St-TFPMB in sensor 4, compared to sensor 3 (0% St-TFPMB), triggered more stable potential readings with a relatively higher slope of 45.5 mV decade^−1^ where 1.0% St-TFPMB enabled more ion-exchange processes, lower membrane resistance, and faster response time [1]. The addition of 1% CD as a modifier in sensor 5 did not improve the response, compared with sensor 4, Figure 4a. Generally, the sub-Nernstian and small concentration range that is observed for different PVC sensors is due to different reasons: first is the presence of LF species in different forms (LF^±^, LF, LF^+^, LF^2+^) [30,31,32,33]; the second reason is the low solubility of the ion-pair in the plasticizer and in the membrane cocktail which limit the mobility of the ions within the membrane and limit the response [1]; the third reason is the presence of the inner filling solution that causes inwards and outwards migrations that cause the limited concentration range [1].

The response mechanism depends on the ion-exchange process of the sodium ion in the membrane phase ($Na_{mem}^{+}$) of the St-TFPMB in the membrane phase with the LF drug in the aqueous phase ($LF_{aq.}^{+}$); this exchange process is governed by the solubility product of the LF-TPB ion-pair within the PVC membrane [44]. This process takes place on the outer solution/membrane interface, which caused a potential difference at this interface, Figure 3a, 3b. The potential difference at the inner filling solution/membrane interface is constant since the composition of inner filling solution and membrane is the same; the resulting potential difference between the two interfaces depends on the concentration of LF^+^ in the outer aqueous solution, and the solubility of the ion-pair in the membrane, neglecting the diffusion potential effect [44,49].

### 3.2. Effect of the Carbon Paste Composition

Optimization of CPEs composition was the base for attaining highly responsive sensors. Thus, the effects of the carbon paste composition as well as the type, and amount of the plasticizer on the potential characteristics of the sensor were investigated. Six CPEs (sensors 6–11) were formulated from Gr, rGO, and MWCNTs as carbon sources, TCP or DOP as plasticizer; St-TFPMB as ion exchanger; LF-TPB as lipophilic ion pair, Figure 4b. The suitable (%, *wt*/*wt*) ratio of the modifiers (GO, rGO, MWCNTs), graphite powder, and plasticizers were chosen according to concentration range, detection limit, and slope (Table 1).

In the case of CPEs (electrodes 6–11, Table 1, Figure 4b), both DOP and TCP were tested (sensors 7 and 8); TCP was the plasticizer of choice in terms of the higher slope, the lower limit of detection, and the small response time; this is expected because of the high TCP dielectric constant that eases mobility of ions and facilitates the ions-exchange process within the paste [1,38], with the absence of problems of solubility limitation that existed in previous PVCEs. For more improvement, other modifiers were tested such as MWCNTs (sensor 9), rGO (sensor 10), and GO (sensor 11). The three modifiers enhance the slope to some extent, and the best slope was in the case of GO (sensor 11) as it has a large surface area, high electron transfer capability, good hydrophobicity, and stable chemical properties which may help in increasing the homogeneity within the paste that improve electrochemical properties of sensor [49,50,51]. The mechanism is the same as described for PVCEs but occurs on the graphite pate/outer solution interface; there is no inner filling solution and so no inner interface (Figure 3b). Therefore, ion-exchange process takes place around this interface and the potential difference is measured through this interface only, with neglecting the diffusion potential effect [44,49].

### 3.3. Effect of the PVC Modification on the CPEs Performance

**Figure 5:**
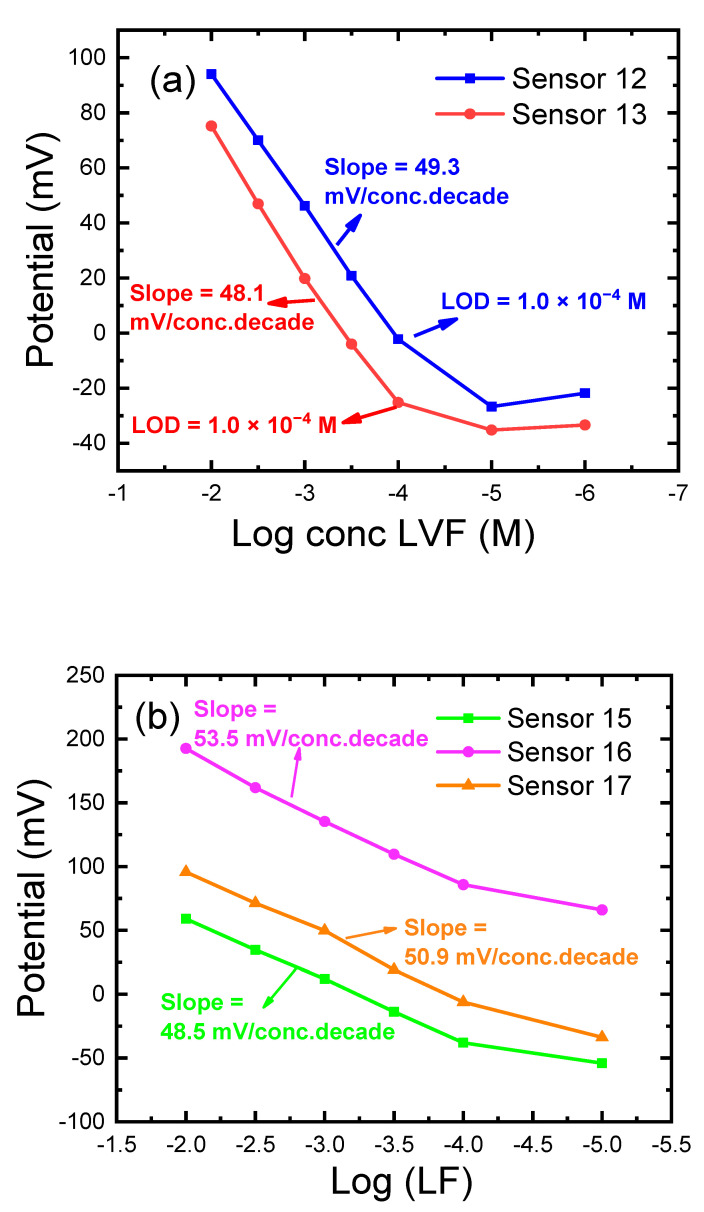
Response characteristics of the prepared electrodes for LF determination: (**a**) calibration curves of the different PVC membrane-coated carbon paste electrodes (C-CPEs); (**b**) Calibration curves of the different PVC/CD plasticized carbon paste electrodes (P-CPEs). The electrode compositions (%, *wt*/*wt*) are given in Table 1. All measurements were at pH 4.1 using acetate buffer at room temperature of 22 ± 2 °C.

Carbon nanomaterials (MWCNTs and GO) were not applied in the case of PVC coated CPEs electrodes (C-CPEs) as they exhibited limited solubility in membrane cocktails. Instead, we prepared C-CPEs (electrodes 12 and 13, Table 1, Figure 3c,d and Figure 5) to advantage the properties of MWCNTs and GO in PVC sensors, Figure 2. Coating a PVC membrane layer (sensor 4) on the CPEs (sensors 9 and 11) resulted in the construction of sensors 12 and 13, respectively, Figure 3c,d and Figure 5. These sensors exhibited a better slope of 50.2 mV decade^−1^ than previous PVCE and CPEs. Moreover, this design was characterized by ease of preparation, and there was no need for water-soluble surfactants which could enhance water penetration [45]. The mechanism is ion-exchange-dependent, controlled by K_sp_ of the LF-TPB. The major difference is the presence of PVC coating that may facilitate the process of ion exchange from and to the aqueous solution, which caused improved slope and selectivity, Figure 3c,d.

**Figure 6:**
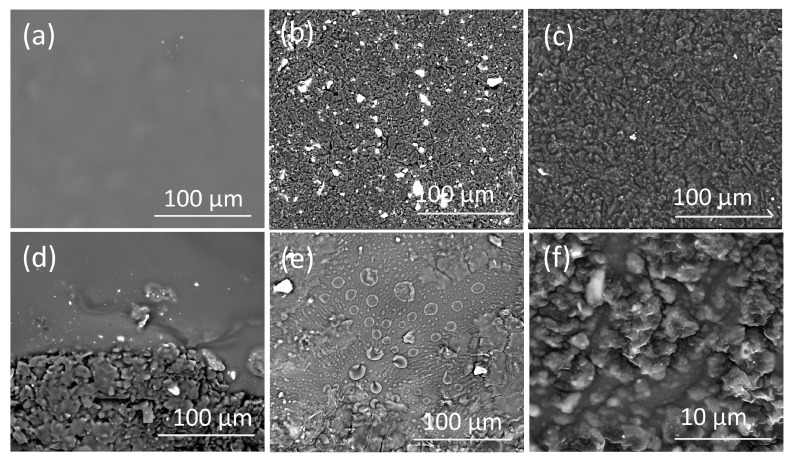
SEM image of the prepared electrodes: (**a**) PVC membrane of sensor 3 (PVCEs), (**b**) carbon paste of sensor 7 (CPE, 47%Gr), (**c**) carbon paste of Sensor 11 (CPE, 44%Gr + 3%GO), (**d**) PVC membrane coated on carbon paste electrode of sensor 13 (C-CPE), (**e**,**f**) PVC plasticized carbon paste electrode of sensor 16 (P-CPE). The electrode compositions (%, *wt*/*wt*) are given in Table 1.

P-CPE (electrodes 14–17, Table 1, Figure 5) were prepared using different modifiers including St-STFPB, ion-pair, and CD, and filled the insulin syringe. The existence of the ion-pair increased the slope from 43.3 in sensor 14 to 48.5 mV decade^−1^ in sensor 15; this confirms the role of the ion-pair as sensing materials in the sensor. The addition of CD in sensor 16 improved the slope to the best ever reported value (53.5 mV/decade^−1^) in the potentiometric determination of LF, Figure 6. This could be attributed to that CD acts as a neutral scavenger that can catch the large drug cationic molecules through H- bonding and host–guest interaction controlled by size effect [52]. Higher amounts of ion-pair in sensor 17 caused a lower slope. Other modifiers such as GO, rGO, and MWCNTs were not used as they exhibited results of bad reproducibility. The mechanism is the same as that of sensor 11 with improved ion pair solubility. Sensors 11, 13, and 16 of improved characteristics were selected for the upcoming studies. The same mechanism applied for P-CPE can be applied here.

### 3.4. Surface Morphology of the PVC Modified CPEs

FE-SEM was used to examine the morphology of the PVCE (sensor 3), the CPE (sensor 7), the C-CPE (sensor 13), and the P-CPES (sensor 16) electrodes. As shown in Figure 6a, the PVC membrane surface is not very smooth, but it has no pores. The SEM image (Figure 6b) shows that the CPEs exhibits granular microstructures. Figure 6c displays the SEM image of the CPEs with GO as carbonaceous nanomaterials mixed with graphite. The presence of GO facilitates the dispersion of LF-TPB within graphite paste, which may be due to the presence of polar carboxyl and hydroxyl groups in the GO that facilitated the hydrogen bonding with the ion pair and facilitate its dispersion and dissolution [53,54,55]. This was confirmed by the absence of white points (LF-TPB particles) in the case of sensor 11, and by the results of SEM-EDX; this lower number of white dots in the case of CPE (sensor 11) and P-CPE (sensor 16) ensures the improved solubility of the ion pair within these electrodes’ paste in comparison with other types, which causes homogeneity of the paste and accounts for the best responses. SEM analysis along with electrochemical measurements confirm that the P-CPE (sensor 16, Figure 6e,f) rationalizes the best response.

### 3.5. Morf Test to Study Water Penetration for the Different Sensors

**Figure 7:**
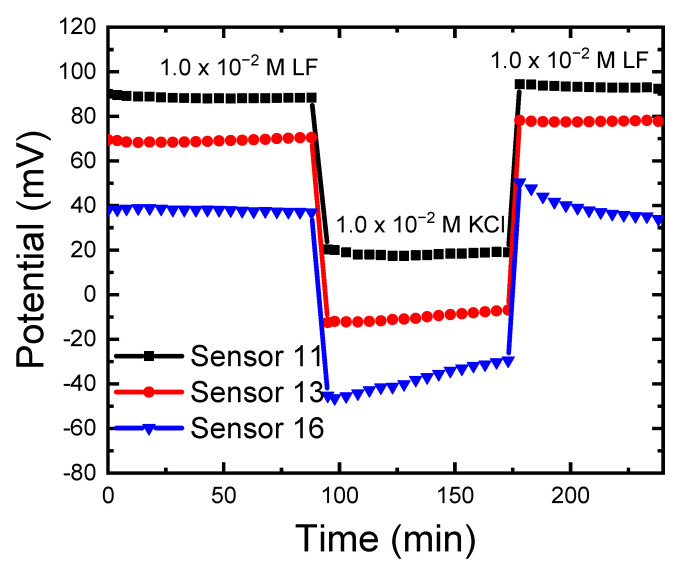
Morf inner water layer test for carbon paste electrode (CPE, sensor 11); PVC membrane coated on carbon paste electrode (C-CPE, sensor 13); PVC/CD plasticized carbon paste electrode (P-CPE, sensor 16). Potentials of the electrodes were recorded in a solution containing the primary ion (1.0 × 10^−2^ M LF) for one hour, then in a solution containing an interfering ion (1.0 × 10^−2^ M KCl) for one hour, and then changing back to the LF solution again. The electrode compositions (%, *wt*/*wt*) are given in (Table 1). All measurements at pH 4.1 using acetate buffer at room temperature of 22 ± 2 °C.

The water layer test also called the “Morf Test” is a crucial validation step of solid-contact ion-selective electrodes (P-CPEs). This test can confirm or contest the claim that the P-CPEs electrode is indeed a genuine solid contact electrode without an aqueous film between the PVC membrane and its solid contact with the carbon paste. Thus, the Morf test was performed to test water insertion between the different phases and between the PVC phase and CPE phase in the different constructed sensors. This test accounts for the response time variation, the stability and reproducibility of measurements, the lifetime, the concentration range, and the detection limit [56]. As in Figure 7, the highest potential drift was observed for P-CPES (sensor 16) followed by lower drift in the case of C-CPE (sensor 13), and the lowest drift was in the case of the CPE (sensor 11). SEM images of the different sensors confirmed the results of the Morf test, which were expected due to the higher probability of water insertion between the PVC phase and carbon paste phase in the case of sensors 13 and 16 [44]. In sensor 16, the rough morphology of the surface became smooth due to the absorption of water; this will affect the lifetime of the sensor [44,45]. A self-assembled lipophilic monolayer with a redox-active component may compensate for this problem [44].

### 3.6. Effect of pH

**Figure 8:**
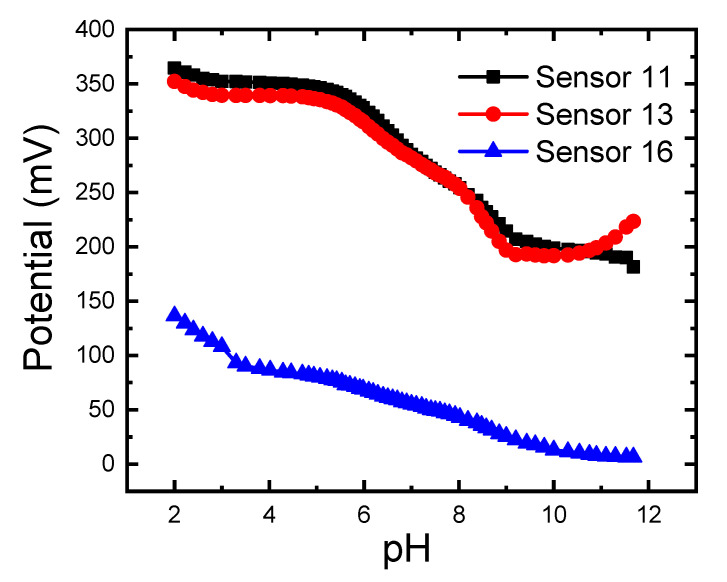
Effect of pH on the response of carbon paste electrode (CPE, sensor 11); PVC membrane coated on carbon paste electrode (C-CPE, sensor 13); PVC/CD plasticized carbon paste electrode (P-CPE, sensor 16) immersed in 10^−3^ M LF. The electrode compositions (%, *wt*/*wt*) are given in (Table 1). All measurements were performed at room te mperature of 22 ± 2 °C.

The pH effect on the potential of sensors (11,13,16) showed variation in potential with pH (Figure 8). The continuous decrease in potential with pH increase is due to the decrease in the number of protons (H+), the higher amount of hydroxide at high pH values, and as a result the presence of the different LF species at the different values. This behavior was observed earlier for LF [31], and ciprofloxacin [57]; it can be understood in terms of the presence of many LF forms (LF^±^, LF, LF^+^, LF^2+^, and LF^−^) that can exist with different ratios at different pH [31,32,33]. Therefore, measurement solutions should be buffered to control species existence with a certain ratio to expect and obtain a confident response of the electrode. We chose pH 2.2 and 4.1 to study the sensor response toward cationic species of LF. At low pH values, LF exists as LF^+^ or LF^2+^, with a low amount in the LF^±^ form [31,32,33]. At pH = 2.2, LF in sample solution exists as LF^2+^ as expected and confirmed by the slope (Table 2). However, the sensor super-Nernstian response at pH 2.2 can be caused by the H^+^-interference [57,58]. Abdel-Haleem et al. and Psomas reported that the best pH for potentiometric measurement of LF is 4.1 due to its existence in its monocationic form in the highest ratio with the coexistence of minimum amount of other LF species [31,32,33]; this also was reported for ciprofloxacin [57]. At pH = 4.1, LF^+^ form dominates, and the sensors sub-Nernstian responses in Table 2 is due to the presence of other LF forms (LF^2+^ and LF^±^), and due to the H-bond formation [57,58]. Now, it is assisted that the LF^2+^ species dominate at pH 2.2 as a major species, while LF^+^ dominates as the major type at pH 4.1; LF^±^ species predominate at pH 7.2. This is the same behavior, as ciprofloxacin [52]. In basic solutions, LF exists in its anionic form.

### 3.7. Selectivity and Interference Study

**Figure 9:**
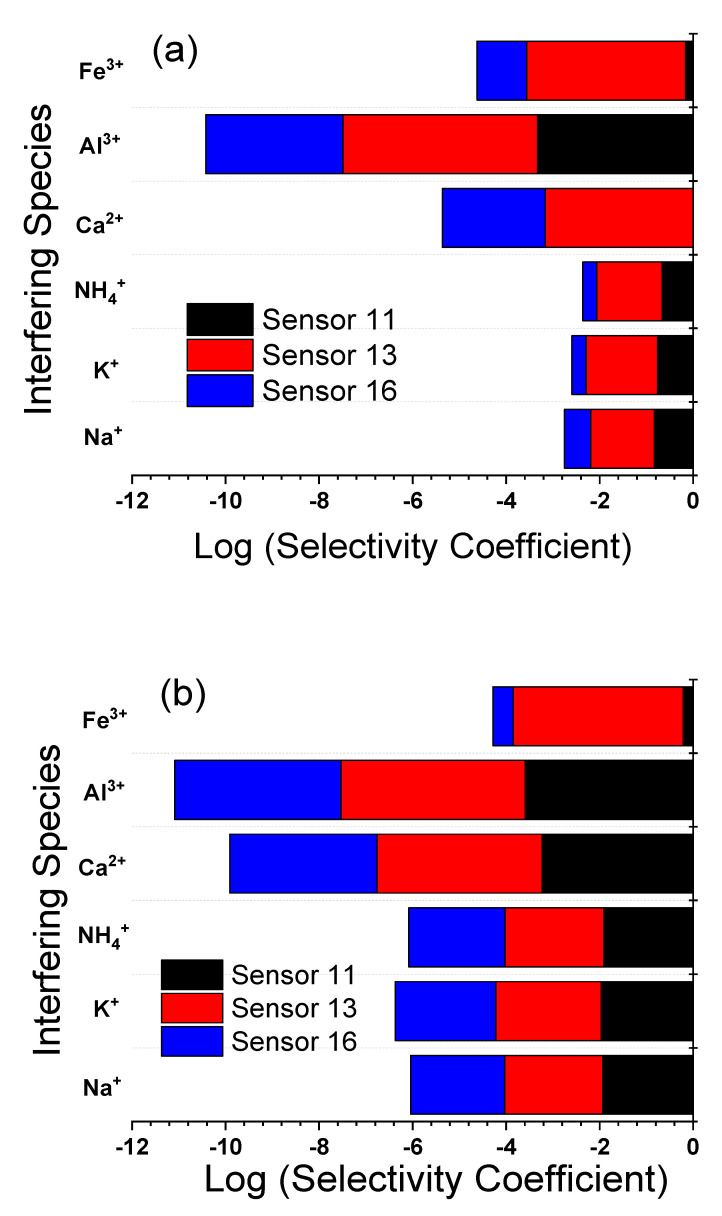
Log selectivity coefficient, ${\log\;K}_{{\mathrm{LF}}^{2+},J^{+z}}^{\mathrm{pot}.}$, determined by using SSM and MPM for a carbon paste electrode (CPE, sensor 11); PVC membrane coated on a carbon paste electrode (C-CPE, sensor 13); PVC/CD plasticized carbon paste electrode (P-CPE, sensor 16) at room temperature of 22 ± 2 °C: (**a**) at pH 2.2; (**b**) at pH 4.1. The electrode compositions (%, *wt*/*wt*) are given in (Table 1).

**Figure 10:**
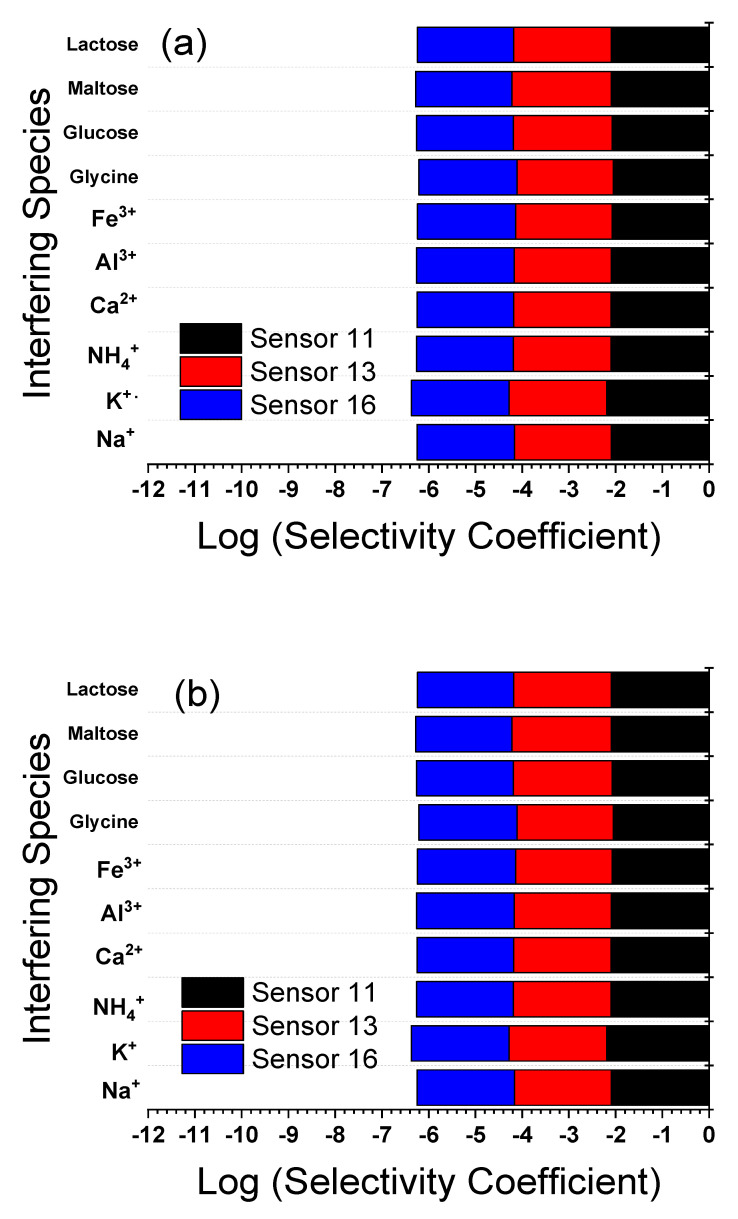
Log selectivity coefficient, ${\log\;K}_{{\mathrm{LF}}^{2+},J^{+z}}^{\mathrm{pot}.}$, determined by using MPM for carbon paste electrode (CPE, sensor 11); PVC membrane coated on carbon paste electrode (C-CPE, sensor 13); PVC/CD plasticized carbon paste electrode (P-CPE, sensor 16) at room temperature of 22 ± 2 °C: (**a**) at pH 2.2; (**b**) at pH 4.1. The electrode compositions (%, *wt*/*wt*) are given in (Table 1).

Since the sensor will be used for the determination of LF in pharmaceutical, serum, and urine samples, some potentially coexistent interferents such as Na^+^, K^+^, Ca^2+^, Fe^3+^, amino acids such as glycine, sugars such as glucose and maltose were examined to evaluate their interference in the determination of LF. It can be deduced from Table 3 and Table 4 that none of these compounds, except Fe^3+^, significantly interfered with the testing of LF (signal change below 5%) in the presence of the same concentration of the different interfering species in case of SSM, up to three-fold concentrations of interfering species in case of MPM, suggesting that the developed method exhibited excellent selectivity for the determination of LF. It is worth mentioning that the selectivity for LF over interfering ions is dependent on the process of ion exchange at the electrode/sample interface, the ion’s mobility at the interface, and the hydrophobic interactions between the analyte ions and electrode surface [1]. Figure 9 and Figure 10, and Table 3 and Table 4 show the results of sensors 11, 13, and 16 at selected pH values using SSM and MPM, over glycine, sugars, and some common inorganic cations which may present in pharmaceuticals. The data in Figure 9 and Figure 10, and Table 3 and Table 4 exhibited high selectivity for LF over the different interferents; only ferric ion exhibited some interference due to the probability of formation of a strong complex with LF, as shown by the ciprofloxacin of similar structure [58]. Since the selectivity of the different sensors is comparable, this ensures that selectivity is a function of the electrode composition and not the electrode physical form [45]. Additionally, Sutter et al. reported the same conclusion for electrodes made of methacrylate copolymer membrane electrode and poly-n-octyl thiophene electropolymerized polymer [56].

### 3.8. Response, Lifetime, and Reversibility

**Figure 11:**
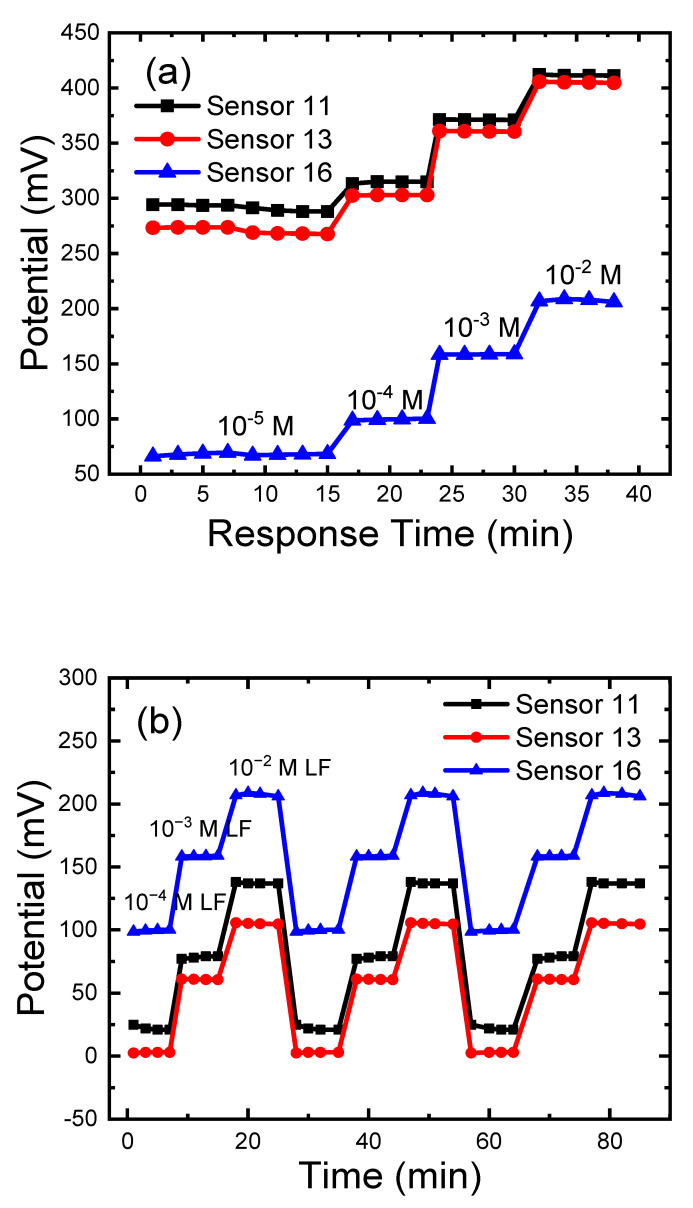
Carbon paste electrode (CPE, sensor 11); PVC membrane coated on carbon paste electrode (C-CPE, sensor 13); PVC/CD plasticized carbon paste electrode (P-CPE, sensor 16): (**a**) response time was monitored by recording the electrodes’ potential in 10^−5^ M, 10^−4^ M, 10^−3^, 10^−2^ LF solution. (**b**) reversibility was followed by recording the electrodes’ potential in 10^−4^ M, 10^−3^, 10^−2^ LF solution. All measurements at pH 4.1 using acetate buffer at room temperature of 22 ± 2 °C. The electrode compositions (%, *wt*/*wt*) are given in (Table 1).

Response time was known to be the time needed to reach about 90% of the final equilibrium potential; it can be measured by soaking the sensor in the different LF solutions (10^−2^–10^−5^ M). All sensors exhibited short response times of less than 1 min, Figure 11a. The faster response time, especially in case of low concentrations, can be attributed to the absence of water film in the case of sensor 11, followed by the response time of sensors 13 and 16; the thickness of the sensing layer is the lowest in case of sensor 11 followed by sensor 13 and 16. Finally came the PVCEs which have the largest sensing layer thickness [56]. The lifetime lies within 5 days for sensor 16, and 4 days for sensors 11 and 13; this lifetime is extended over that reported earlier due to the modification by PVC by coating or insertion in the paste. The shorter lifetime of sensor 16 is due to the water film between PVC and paste layers, which may facilitate ion pair solubility and shorten lifetime, as confirmed by the Morf test [44,45]. The water penetration ability is largest in the case of sensor 16 because the larger surface area of the electrode is in contact with the solution, and the PVC layer in the case of sensor 13 will need a longer contact time for effective water penetration. This lower amount of penetrated water in the case of CPE and C-CPE, as confirmed by the Morf test, caused a longer lifetime of the sensors. The reversibility of the LF sensors (11,13 and 16) was tested by immersing the electrode in LF solutions of concentrations 10^−3^ and 10^−4^ M, Figure 11b, that confirm satisfactory reversibility.

### 3.9. Analytical Applications

Due to the wide applications of LF in treating several diseases and its serious side effects, numbers of electrochemical biosensors have been reported for electrochemical determination of LF using for example a boron-doped diamond electrode [59], glassy carbon electrode (GCE) [60,61], and molecularly imprinted polymers modified with gold nanoparticles [62]. All these methods depend mainly on voltametric determination which needs sophisticated instruments, expensive nanomaterials such as gold and requires multiple sample preparation steps. To overcome these restrictions, potentiometric method-based CPEs have been developed because they are rapid, simple, and cost-effective alternatives. The change in the ratios of the different electrode constituents directly affects the slope, selectivity, life and response times, and detection limit of the proposed sensor [46]. LF can form different species in aqueous solution: the zwitterionic LF^±^, the neutral LF, the monocationic LF^+^, the dicationic LF^2+^, and the anionic LF^−^ species [30,31,32,33]; so it is significant to detect the existing species that dominates at the measurement pH. As a primary value, pH 4.1 was chosen to assist the existence of mono-cationic species as the main species [30,31,32,33] and to study the effect of electrode composition. It is worth mentioning that the highest permissible LF dose for adult humans should not exceed 1500 mg/kg/day, which is very close to the lethal dose (~1.800 mg/Kg (9.15 × 10^−4^ M)) [56]. To cover this range, direct potentiometry was performed and it showed good recoveries for LF determination in their pure solutions, pharmaceutical formulations (Levoxin^®^ 500 mg), spiked samples of urine and serum using sensors 11, 13 and 16 at the different pH values (Table 5 and Table 6); the results show accuracy and precision, where recovery ranges were 93.0–101.0 and 87.1–101.3, at pH 2.2 and 4.1, respectively. Precision is confirmed since all standard deviation values are lower than one.

By comparing with LF sensors previously as reported potentiometric sensors [30,31], the response characteristics of the best sensors constructed in this work show significant improvemtn in the different response characteristics (Table 7). AbdelGhani et al. [31] reported a slope of 51.5 mV/conc.decade for CPE at a 10^−2^–10^−4^ M concentration range with a 5.0 × 10^−5^ M detection limit; this work improved the slope to 51.5 mV/conc.decade in the same range with a comparable detection limit [31]. Rizka et al. [30] reported a selectivity coefficient logarithm, in the case of Ca^2+^, of −2.29 and −1.39; this work improved these values by about two orders of magnitude. These improvement in selectivity, detection limit and slope is due to the implementation of the PVC layer in case of C-CPE and P-CPES. In another study, Rkik et al. [59] electrochemically determine LF in biological samples using a boron-doped-diamond electrode with LOD of 2.88 × 10^−6^ M [59]. Radi and El-Sherif [60] electrochemically determine LF in diluted urine samples using a GCE electrode with cyclic and square-wave voltammetry. The detection limit of this method was calculated to be 5.0 × 10^−9^ M [60]. Tang et al. [61] used Ag NPs and an electrospun CeO_2_-Au composite nanofiber-modified GCE electrode for determination of LF by applying scanning electron microscopy and electrochemical methods. The sensor achieved a linear concertation range of 0.03–10 × 10^−6^ M with a LOD as low as 0.01 × 10^−6^ M [61]. Wang et al. [62] developed molecularly imprinted polypyrrole–graphene–gold nanoparticles modified electrode-based electrochemical sensor for LF determination. This sensor showed a linearly with the concentration of LF in the range from 1.0 to 100 × 10^−6^ M with an LOD of 0.53 × 10^−6^ M [62].

## 4. Conclusions

Levofloxacin (LF) drug is an antibiotic medication widely used to treat several types of bacterial infections, urinary tract infections, chronic prostatitis, and some types of gastroenteritis. In this work, a rapid and low-cost method for the development of electrochemical sensors based on carbon paste electrodes (CPEs) modified with polyvinyl chloride (PVC) was proposed. The modification CPEs by the coating of a PVC membrane on the CPE surface (C-CPE) or by incorporating PVC/cyclohexanone paste in the formulation of the CPE paste (P-CPEs referenced as P-CPE). LF has several species at different pH, so the buffer is a prerequisite to control the predominant species. The different sensors exhibited a selective response toward LF in the presence of all the expected interfering ions except ferric ions which show little interference. The fast response time of the developed sensors facilitates the use of these sensors in routine analysis with simple instrumentation. The modified CPEs were used for the detection of LF in serum, urine, pharmaceutical formulation. Under the optimized experimental conditions, the best carbon paste electrode exhibited a sub-Nernstian response of 49.3 mV decade^−1^ within the LF concentration range from 1.0 × 10^−2^ M to 1.0 × 10^−5^ M, with a detection limit of 1.0 × 10^−5^ M. Implementation of the PVC layer in the case of the coated carbon paste electrode (C-CPE)and plasticized carbon paste electrode (P-CPE) improved the slope, detection limit, and the selectivity, especially ferric ions which can form a complex with LF, and it may be present in physiological fluids. The selectivity against different interfering species (Na^+^, K^+^, NH^4+^, Ca^2+^, Al^3+^, Fe^3+^, Glycine, Glucose, Maltose, Lactose) were significantly improved by one to three orders of magnitudes; by comparison, the selectivity coefficient (${\log\;K}_{{\mathrm{LF}}^{2+},{\mathrm{Fe}}^{+3}}^{\mathrm{pot}.}$) was improved from −0.16 in CPE to −3.40 and −1.06 in the case of C-CPE and P-CPE, respectively. The developed sensors exhibited good reversibility and were applied for the determination of the drug in spiked physiological samples with good recovery values.

## Figures and Tables

**Table 1:** Percent compositions (*wt*/*wt*%) of the different sensors and their response properties using acetate buffer at pH 4.1 at room temperature of 22 ± 2 °C.

Sensor	Percent Compositions (*wt*/*wt*%) (*wt*/*wt*)%					Response Characteristics			
	IP	PVCM	Gr	Additive	Plasticizer	C.R. (M)	LOD (M)	Slope	RSD (%)
1	-	33.0	-	1.0 St-TFPMB	66.0 TCP	10^−2^–10^−5^	1.0 × 10^−5^	50.0	0.43
2	-	33.0	-	1.0 St-TFPMB	66.0 DOP	10^−2^–10^−5^	1.0 × 10^−5^	47.5	2.11
3	5.0	31.7	-	-	63.3 DOP	10^−2^–10^−4^	2.1 × 10^−5^	43.0	3.95
4	5.0	31.3	-	1.0 St-TFPMB	62.7 DOP	10^−2^–10^−4^	2.5 × 10^−5^	45.5	2.26
5	5.0	26.0	-	1.0 St-TFPMB + 1.0 CD	67.0 DOP	10^−2^–10^−4^	7.8 × 10^−5^	43.0	2.33
6	-	-	49.5	1.0 St-TFPMB	49.5 TCP	10^−2^–10^−5^	1.0 × 10^−5^	51.5	3.7
7	5.0	-	47.0	1.0 St-TFPMB	47.0 TCP	10^−2^–10^−4^	1.0 × 10^−5^	46.5	0.7
8	5.0	-	47.0	1.0 St-TFPMB	47.0 DOP	10^−2^–10^−5^	8.4 × 10^−5^	44.0	4.1
9	5.0	-	44.0	1.0 St-TFPMB + 3.0 MWCNTs	47.0 TCP	10^−2^–10^−5^	1.0 × 10^−5^	46.5	1.5
10	5.0	-	44.0	1.0 St-TFPMB + 3.0 rGO	47.0 TCP	10^−2^–10^−5^	1.0 × 10^−5^	47.7	3.7
11	5.0	-	44.0	1.0 St-TFPMB + 3.0 GO	47.0 TCP	10^−2^–10^−5^	1.0 × 10^−5^	49.3	4.8
12	5.0	Coated*	44.0	1.0 St-TFPMB + 3.0 MWCNTs	47.0 TCP	10^−2^–10^−4^	1.0 × 10^−4^	48.1	0
13	5.0	Coated*	44.0	1.0 St-TFPMB + 3.0 GO	47.0 TCP	10^−2^–10^−4^	1.0 × 10^−4^	50.2	4.2
14	-	-	30.3	1.0 St-TFPMB	30.0 TCP + 38.7 PVCP	10^−2^–10^−4^	1.0 × 10^−5^	43.3	0
15	5.0	-	27.8	1.0 St-TFPMB	30.0 TCP + 36.2 PVCP	10^−2^–10^−4^	5.6 × 10^−5^	48.5	6.8
16	5.0	-	27.0	1.0 St-TFPMB + 1.0 CD	30.0 TCP + 36.0 PVCP	10^−2^–10^−4^	6.3 × 10^−5^	53.5	4.4
17	10.0	-	24.5	1.0 St-TFPMB + 1.0 CD	30.0 TCP + 33.5 PVCP	10^−2^–10^−4^	2.8 × 10^−5^	50.9	4.5

IP: ion-pair (LF-TPB); PVCM: PVC contents used in the formulation of PVC membrane of the PVCEs; Gr: Graphite; St-TFPMB: sodium tetrakis (trifluoromethyl)phenyl borate as an ion exchanger; CD: cyclodextrin as a modifier; MWCNTs; multi-walled carbon nanotubes as carbon modifier; rGO: reduced graphene oxide as carbon modifier; TCP: tricresyl phosphate as a plasticizer, PVCP: PVC liquid paste used in the preparation of the P-CPEs; DOP: dioctyl phthalate as a plasticizer, LOD: limit of detection in M; slope in mV/conc.decade; RSD: relative standard deviation for three measurements; *Coated: PVC membrane of composition in sensor 4 coated on the CPE. Relative standard deviation (RSD) calculations were based on at least three measurements.

**Table 2:** Effect of the pH on the response characteristics of the optimized sensors.

Sensors	pH	Slope	Detection Limit	Linear Dynamic Range	RSD
		mV/conc. Decade	M	M	%
CPE (Sesnor 11)	2.2	35.4	3.16 × 10^−5^	10^−2^–10^−5^	0.2
	4.1	49.3	1.0 × 10^−5^	10^−2^–10^−4^	4.8
C-CPE (Sesnor 13)	2.2	41.8	2.5 × 10^−5^	10^−2^–10^−4^	5.14
	4.1	50.2	1.0 × 10^−4^	10^−2^–10^−4^	4.2
P-CPE (Sesnor 16)	2.2	32.0	3.98 × 10^−5^	10^−2^–10^−4^	4.47
	4.1	53.5	6.3 × 10^−5^	10^−2^–10^−4^	4.4

Relative standard deviation (RSD%) calculations were based at leasr on three paraell measuremnts.

**Table 3:** Selectivity coefficient log, ${\log\;K}_{{\mathrm{LF}}^{2+},J^{+z}}^{\mathrm{pot}.}$ , for the selected sensors at room temperature of 22 ± 2 °C at pH 2.2.

Interferent	CPE (Sensor 11)		C-CPE (Sensor 13)		P-CPE (Sensor 16)	
	SSM	MPM	SSM	MPM	SSM	MPM
Na^+^	−0.83	−2.09	−1.36	−2.07	−0.56	−2.09
K^+^	−0.76	−2.19	−1.53	−2.09	−0.30	−2.09
NH_4_^+^	−0.66	−2.10	−1.40	−2.09	−0.30	−2.07
Ca^2+^	−−2.46	−2.10	−3.16	−2.08	−2.2	−2.07
Al^3+^	−3.33	−2.09	−4.16	−2.08	−2.93	−2.09
Fe^3+^	−0.16	−2.07	−3.40	−2.07	−1.06	−2.10
Glycine	-	−2.05	-	−2.06	-	−2.10
Glucose	-	−2.07	-	−2.12	-	−2.07
Maltose	-	−2.09	-	−2.13	-	−2.06
Lactose	-	−2.09	-	−2.09	-	−2.06

**Table 4:** Selectivity coefficient log, ${\log\;K}_{{\mathrm{LF}}^{+},J^{+z}}^{\mathrm{pot}.}$ , for the selected sensors at pH 4.1 at room temperature of 22 ± 2 °C.

Interferent	CPE (Sensor 11)		C-CPE (Sensor 13)		P-CPE (Sensor 16)	
	SSM	MPM	SSM	MPM	SSM	MPM
Na^+^	−1.93	−2.09	−2.10	−2.07	−2.01	−2.09
K^+^	−1.96	−2.19	−2.26	−2.09	−2.15	−2.09
NH_4_^+^	−1.91	−2.10	−2.11	−2.09	−2.06	−2.07
Ca^2+^	−3.23	−2.10	−3.53	−2.08	−3.15	−2.07
Al^3+^	−3.59	−2.09	−3.94	−2.08	−3.56	−2.09
Fe^3+^	−0.21	−2.07	−3.64	−2.07	−0.43	−2.10
Glycine	-	−2.05	-	−2.06	-	−2.10
Glucose	-	−2.07	-	−2.12	-	−2.07
Maltose	-	−2.09	-	−2.13	-	−2.06
Lactose	-	−2.09	-	−2.09	-	−2.06

**Table 5:** Recovery values of the best sensors for the determination of LF using a direct potentiometric method at room temperature of 22 ± 2 °C at pH 2.2. The electrode compositions (%, *wt*/*wt*) are given in (Table 1).

Samples	LF Conc.	CPE (Sensor 11)	C-CPE (Sensor 13)	P-CPE (Sensor 16)
Pure solution	10^−3^ M	100.1 ± 0.1	99.8 ± 0.1	99.7 ± 0.2
	10^−4^ M	100.3 ± 0.2	100.2 ± 0.3	100.1 ± 0.3
Pharm. solution	10^−3^ M	99.8 ± 0.2	95.0 ± 0.3	99.1 ± 0.5
	10^−4^ M	94.4 ± 0.5	93.0 ± 0.5	94.4 ± 0.3
serum solution	10^−3^ M	99.7 ± 0.5	99.5 ± 0.8	98.5 ± 0.6
	10^−4^ M	93.5 ± 0.8	99.6 ± 0.7	99.2 ± 0.3
Urine solution	10^−3^ M	99.2 ± 0.9	101.0 ± 0.7	95.5 ± 0.7
	10^−4^ M	95.5 ± 0.8	94.4 ± 0.9	93.5 ± 0.8

**Table 6:** Recovery values of the best sensors for the determination of LF using the direct potentiometric method method at room temperature of 22 ± 2 °C at pH 4.1. The electrode compositions (%, *wt*/*wt*) are given in (Table 1).

Samples	LF Conc.	CPE (Sensor 11)	C-CPE (Sensor 13)	P-CPE (Sensor 16)
Pure solution	10^−3^ M	99.7 ± 0.1	101.3 ± 0.1	100.1 ± 0.1
	10^−4^ M	99.3 ± 0.1	99.7 ± 0.2	99.2 ± 0.2
Pharm. solution	10^−3^ M	99.0 ± 0.1	89.2 ± 0.5	93. 3 ± 0.3
	10^−4^ M	98.1 ± 0.3	94.4 ± 0.3	100.0 ± 0.2
serum solution	10^−3^ M	98.7 ± 0.7	100.0 ± 0.6	87.1 ± 0.6
	10^−4^ M	95.6 ± 0.4	95.5 ± 0.5	100.0 ± 0.5
Urine solution	10^−3^ M	98.4 ± 0.4	100.0 ± 0.8	100.0 ± 0.7
	10^−4^ M	94.5 ± 0.9	95.5 ± 0.8	100.1 ± 0.8

**Table 7:** Comparison between response characteristics of the best sensors (carbon paste electordes) construcuted in this work and those reportrd by Rizka et al. [30] and AbdelGhani et al. [31]. The electrode compositions (%, *wt*/*wt*) are given in Table 1.

Comparison	This work			Rizka et al. [30]		AbdelGhani et al. [31]	
	CPE (Sensor 11)	C-CPE (Sensor 13)	P-CPE (Sensor 16)	Le-Re	Le-FI	CPE	SPE
Slope, mV/conc. Decade	49.3	50.2	53.5	50.3	48.5	51.5	44.0
Detection limit, M	1.0 × 10^−5^	7.3 × 10^−5^	6.3 × 10^−5^	1.0 × 10^−4^	1.0 × 10^−4^	5.0 × 10^−5^	2.5 × 10^−5^
Linear dynamic range, M	10^−2^–10^−5^	10^−2^–10^−4^	10^−2^–10^−4^	10^−2^–10^−4^	10^−2^–10^−4^	10^−2^–10^−4^	10^−2^–10^−4^
Lifetime, days	14	14	5	-	-	7	7
${\log\;K}_{{\mathrm{LF}}^{+},{\mathrm{Fe}}^{+3}}^{\mathrm{pot}.}$	−0.21	−3.64	−0.43	−1.18	−1.67	−0.66	−1.78
${\log\;K}_{{\mathrm{LF}}^{+},{\mathrm{Ca}}^{+2}}^{\mathrm{pot}.}$	−3.23	−3.35	−3.15	−2.29	−1.39	−3.08	−3.31

## Data Availability

The data presented in this study are available on request from the corresponding author.
